# Microfluidic-assisted silk nanoparticle tuning†

**DOI:** 10.1039/c8na00208h

**Published:** 2018-11-30

**Authors:** Thidarat Wongpinyochit, John D. Totten, Blair F. Johnston, F. Philipp Seib

**Affiliations:** Strathclyde Institute of Pharmacy and Biomedical Sciences, University of Strathclyde 161 Cathedral Street Glasgow G4 0RE UK philipp.seib@strath.ac.uk; Leibniz Institute of Polymer Research Dresden, Max Bergmann Center of Biomaterials Dresden Hohe Strasse 6 01069 Dresden Germany

## Abstract

Silk is now making inroads into advanced pharmaceutical and biomedical applications. Both bottom-up and top-down approaches can be applied to silk and the resulting aqueous silk solution can be processed into a range of material formats, including nanoparticles. Here, we demonstrate the potential of microfluidics for the continuous production of silk nanoparticles with tuned particle characteristics. Our microfluidic-based design ensured efficient mixing of different solvent phases at the nanoliter scale, in addition to controlling the solvent ratio and flow rates. The total flow rate and aqueous : solvent ratios were important parameters affecting yield (1 mL min^−1^ > 12 mL min^−1^). The ratios also affected size and stability; a solvent : aqueous total flow ratio of 5 : 1 efficiently generated spherical nanoparticles 110 and 215 nm in size that were stable in water and had a high beta-sheet content. These 110 and 215 nm silk nanoparticles were not cytotoxic (IC50 > 100 μg mL^−1^) but showed size-dependent cellular trafficking. Overall, microfluidic-assisted silk nanoparticle manufacture is a promising platform that allows control of the silk nanoparticle properties by manipulation of the processing variables.

## Abstract 2

Silk is now making inroads into advanced pharmaceutical and biomedical applications.

## Graphical abstract

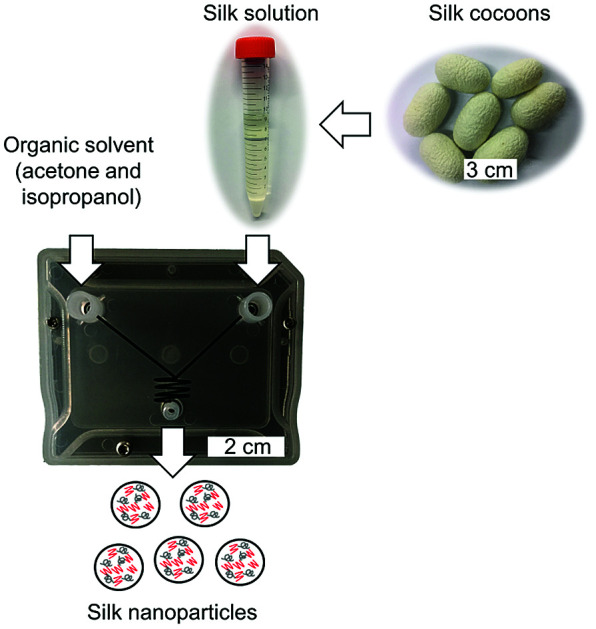

## Introduction

Everyday silk from the silk moth (*Bombyx mori*) is used both in the textile industry and in medical applications (most notably as a surgical suture material).^1^ Over the past 30 years, a renewed interest has grown in the silk biopolymer for use in medical devices, including its recently approved use as a surgical scaffold for supporting and repairing soft-tissue damage in humans.^2^ Silk is consistently viewed as a promising biopolymer for biomedical applications across a broad range of applications.^1^

Silk has several important and exploitable characteristics, including (i) excellent mechanical properties, (ii) a long-term track record of its safe use in humans, (iii) broad biocompatibility and biodegradability, (iv) mild aqueous processing conditions, and (v) the ability to stabilize and protect therapeutic payloads (*e.g.*, proteins and small molecular drugs).^3,4^ In addition, a reversed engineered silk solution can be processed into numerous material formats, including hydrogels, scaffolds, films, microspheres, and nanoparticles (reviewed in^5,6^). For these reasons, silk nanoparticles are emerging as interesting carriers for drug delivery and are now often proposed for solid tumor drug targeting.^7–10^ Silk nanoparticles can be refined—for example, by surface decorating with polyethylene glycol (PEG)—to further tailor their performance by improving their colloidal stability and tuning their immune recognition.^8,11^ Both native and PEGylated silk nanoparticles have demonstrated high drug loading efficacy, pH-dependent drug release, and selective degradation by protease enzymes as well as by *ex vivo* lysosomal enzymes.^12^

Silk nanoparticles can be manufactured by a broad spectrum of methods (reviewed in ref. 6 and 13), including poly(vinyl alcohol) blending (size range 300 nm to 10 μm),^14^ emulsification (170 nm),^15^ capillary microdot printing (25–140 nm),^16^ salting out (486–1200 nm),^17^ supercritical fluid technologies (50–100 nm),^18^ ionic liquid dissolution (180 nm),^19^ electrospraying (59–80 nm),^20^ vibrational splitting of a laminar jet (up to 400 μm),^21^ electric fields (200 nm to 3 μm),^22^ milling technologies (200 nm),^23^ and organic solvent desolvation (35–170 nm).^7,8,24,25^ Among these methods, the desolvation method for manufacture of silk nanoparticles is a robust and reproducible technique for the production of stable and uniform nano-sized particles. This method involves mixing an aqueous silk solution with a water-miscible organic solvent (*e.g.*, methanol, isopropanol, acetone, *etc.*) to cause the nanoprecipitation of silk and the formation of silk nanoparticles. However, the current desolvation methods used to generate silk nanoparticles are time-consuming batch processes that allow little in-process control for tuning nanoparticle characteristics such as particle size. The ability to control the particle size and polydispersity of nanoparticles designed for drug delivery applications is important, as these particle attributes affect performance factors such as loading capacity (and thus drug dosage), targeting capabilities, cellular uptake, and both whole body and cellular pharmacokinetic characteristics.^26^

Over the past decade, remarkable progress has been made in the development of microfluidic-based fluid handling systems that can be applied to particle production for drug delivery applications (*e.g.*, lipid, solid, tuned shape, *etc.*). Microfluidics enable the precise manipulation of liquids that allow the control of process parameters, such as the total flow rate, flow rate ratios between different phases, particle geometry, drug loading, *etc.*^27–29^ Nevertheless, despite the advantages of microfluidics, few studies have exploited this technology to generate silk particles. Some approaches have included glass capillary-based microfluidics (resulting in particles 145–200 μm in size),^30^ double junction microfluidics (10–200 μm particles)^31^ and single and double T-junction droplet microfluidics (colloids 5–80 μm).^32^ However, these previous studies produced micro-sized particles that are too large in size for use as carriers in many drug delivery applications (*e.g.*, tumor targeting following intravenous dosing, endocytic uptake, intracellular trafficking, *etc.*).

The aim of the current study was therefore to manufacture silk nanoparticles by desolvation using the NanoAssemblr™ microfluidic setup. We investigated the impact of several process parameters, such as the total flow rate, flow rate ratios (*i.e.*, aqueous to organic solvent), and organic solvent choices (acetone and isopropanol) on silk nanoparticle physical characteristics (*e.g.*, yield, particles size, polydispersity, zeta potential, stability, secondary structure, and morphology). We manufactured bespoke silk nanoparticles to demonstrate the impact of silk nanoparticle size on uptake and intracellular trafficking.

## Materials and methods

### Manufacturing of silk nanoparticles by microfluidics

*Bombyx mori* cocoons were cut into approximately 5 × 5 mm pieces and degummed by boiling in 0.02 M Na_2_CO_3_ for 60 min. The degummed fibers were then rinsed in ultrapure water and air-dried. The dry fibers were dissolved in 9.3 M LiBr solution at 60 °C for up to 4 h and subsequently dialyzed (molecular weight cut off 3500 g mol^−1^) against ultrapure water for 48 h to remove the LiBr salt. The resulting aqueous silk solution was cleared by centrifugation. A visual protocol format showing reverse engineering of silk cocoons is available.^25^

**Fig. 1:**
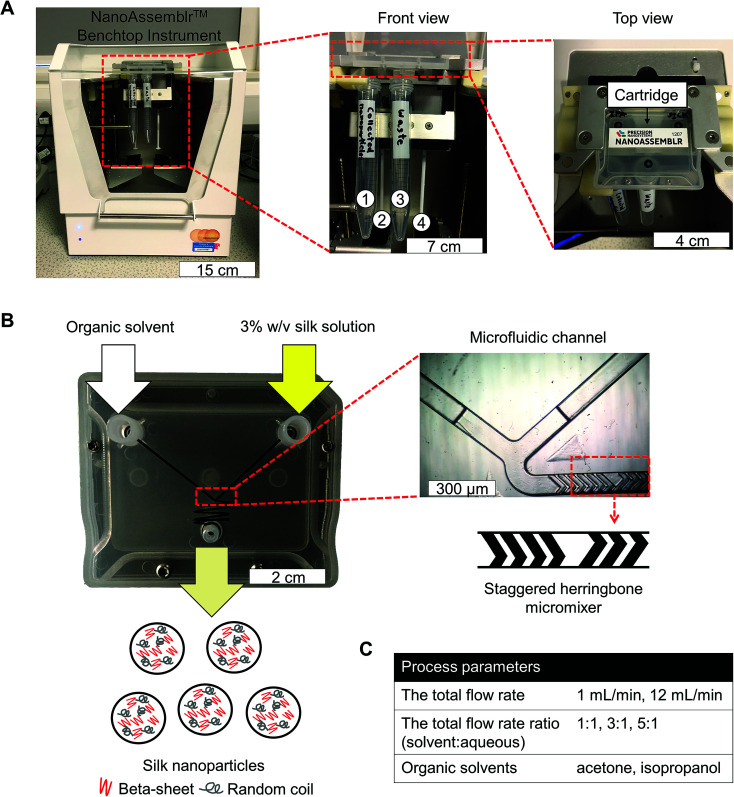
Schematic of silk nanoparticles manufacture using a microfluidic cartridge coupled with a NanoAssemblr™ benchtop instrument. (A) NanoAssemblr™ benchtop instrument with major components and arrangement. At front view, number 1 and 3 identify collection tubes for nanoparticles and waste, respectively. Number 2 and 4 are syringes containing an organic solvent and a 3% w/v silk solution, respectively. (B) Organic solvent and the silk solution are pumped into two inlets and rapidly three dimensional mixed, which leads to silk nanoparticle formation by nanoprecipitation. The microfluidic cartridge contains a micromixer channel, which is designed as a staggered herringbone structure. (C) The total flow rate, total flow rate ratio, and solvent choice were the process parameters for this study.

Silk nanoparticles were manufactured using a NanoAssemblr™ benchtop instrument version 1.5 (model number: SN: NA-1.5-16) (NanoAssemblr™, Precision Nano-Systems Inc. Vancouver, Canada) equipped with a microfluidic cartridge (product code: NIT0012) (Fig. 1A). A 3% w/v aqueous silk solution and organic solvent (either acetone or isopropanol) were injected into separate chamber inlets, the silk nanoprecipitated in the microfluidic mixer, and the resulting nanoparticles were collected in the outlet (Fig. 1B). The total flow rates of the organic solvent and silk solution were varied from 1 to 12 mL min^−1^, and the flow rate ratio was varied at 1 : 1, 3 : 1 and 5 : 1 (Fig. 1C). The collected silk nanoparticles were centrifuged at 48 400 × *g* for 2 h and the supernatant was aspirated and discarded. The pellet was resuspended in ultrapure water, vortexed, and subsequently sonicated twice for 30 s at 30% amplitude with a Sonoplus HD 2070 sonicator (ultrasonic homogenizer, Bandelin, Berlin, Germany). These centrifugation, washing, and resuspension steps were repeated at least twice more to produce the final silk nanoparticle suspension. The nanoparticles were characterized as detailed below and stored at 4 °C until use.

### The yield of silk nanoparticles

The total volume of the silk nanoparticle stock suspension was determined. Next, several 2 mL Eppendorf tubes were weighed before adding silk nanoparticles (*W*1). The manufactured silk nanoparticles were then added, frozen, and lyophilized overnight. The tubes containing the resulting freeze-dried silk nanoparticles were weighed again (*W*2) to determine the amount of silk nanoparticles and overall yield eqn (1).1
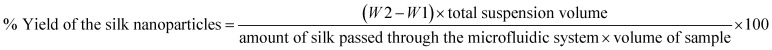


### Silk nanoparticle characterization and stability in water

The particle size, polydispersity index (PDI), and zeta potential of silk nanoparticles in ultrapure water were determined at 25 °C by dynamic light scattering (DLS, Zetasizer Nano-ZS Malvern Instrument, Worcestershire, UK). Particle size was determined using refractive indices of 1.33 for water and 1.60 for protein. The silk nanoparticles were stored in water at 4 °C and 37 °C and the size, PDI, and zeta potential were determined at days 0, 14, 28, 35, and 42. All measurements were conducted in triplicate.

### Secondary structure measurements of silk nanoparticles

The silk nanoparticle suspension was frozen and then lyophilized overnight. The samples were subjected to secondary structure analysis by Fourier transform infrared (FTIR) spectroscopy (TENSOR II FTIR spectrometer, Bruker Optik GmbH, Ettlingen, Germany). Each measurement was run for 128 scans at a 4 cm^−1^ resolution over the wavenumber range of 400 to 4000 cm^−1^. OriginPro 9.2 Software was used to correct the baseline and peak fit at the amide I region (1595–1705 cm^−1^), based on previous analyses.^33^ Briefly, the amide I region was identified and deconvoluted: 1605–1615 cm^−1^ as side chain/aggregated strands, 1616–1637 cm^−1^ and 1697–1703 cm^−1^ as beta-sheet structure, 1638–1655 cm^−1^ as random coil structure, 1656–1662 cm^−1^ as alpha-helical bands, and 1663–1696 cm^−1^ as turns. The second derivative was applied at the amide I region for peak finding. Gaussian line shapes were used for curve fitting. Overfitting of the data was avoided by fixing the peak full width at half-maximum (FWHM) at 10 cm^−1^.

### Scanning electron microscopy of silk nanoparticles

The morphology of the prepared silk nanoparticles was assessed by scanning electron microscopy (SEM) using a FE-SEM SU6600 instrument (Hitachi High Technologies, Krefeld, Germany) at 5 kV. Samples were pipetted onto a silicon wafer and lyophilized overnight. The specimens were coated with gold (15 nm thickness) using an ACE200 low vacuum sputter coater (Leica Microsystems, Wetzlar, Germany). The SEM images were processed using ImageJ v1.51j8 (National Institutes of Health, Bethesda, MD).^34^

### Manufacture of silk nanoparticles for *in vitro* assays

Silk nanoparticles were manufactured by the automated microfluidic NanoAssemblr™ benchtop instrument, as detailed above. The total flow rate and ratio of isopropanol and 3% w/v aqueous silk solution were varied depending on the formulations: (i) 5 : 1 at 1 mL min^−1^ for 110 nm size and (ii) 5 : 1 at 12 mL min^−1^ for 215 nm size.

### Macrophage responses toward silk nanoparticles

The murine macrophage RAW 264.7 cell line was purchased from ATCC (Manassas, VA, U.S.A.). Cells were cultured in Dulbecco's Modified Eagle Medium (DMEM) (4.5 g glucose, 110 mg sodium pyruvate, 10% v/v FBS), grown in a humidified 5% CO_2_ atmosphere at 37 °C and routinely subcultured every 2–3 days by scraping cells off the flask and replating them at a split ratio of 1 : 10 on tissue culture treated polystyrene (Corning, New York, NY, U.S.A.). For cytotoxicity studies, cells were seeded in 96-well plates at a density of 1.5 × 10^4^ cells per cm^2^ and allowed to recover 24 h. Next, cells were treated with 2.5 to 100 μg mL^−1^ of 110 nm and 215 nm silk nanoparticles. After a 48 h of incubation, cell viability was determined using 3-(4,5-dimethylthiazol-2-yl)-2,5-diphenyltetrazolium bromide (MTT; 5 mg mL^−1^ in phosphate buffered saline (PBS)); 20 μL of MTT was added to each well and cultures were incubated for 5 h. The formazan product was solubilized with 100 μL of dimethyl sulfoxide (DMSO) and absorbance was measured at 570 nm. Untreated control cells represented 100% cell viability.

For tumor necrosis factor alpha (TNF-α) release, cells were seeded in Petri dishes at a density of 1.5 × 10^4^ cells per cm^2^ and allowed to recover overnight. Next, the culture medium was aspirated and replaced with fresh medium containing either (i) 15 ng of lipopolysaccharide (LPS, Sigma-Aldrich, St. Louis, MO, U.S.A.), (ii) 10 μg mL^−1^ and 500 μg mL^−1^ of either 110 nm or 215 nm silk nanoparticles, and (iii) control medium. Cultures were incubated for 24 h and then the medium was collected and centrifuged at 6000 × *g* for 5 min. The supernatants were stored at −80 °C until analysis. Culture supernatants were assayed for mouse TNF-α using a DuoSet ELISA (R&D Systems, Minneapolis, MN, USA) according to the manufacturer's instructions. All measurements were derived from three biological replicates.

### Labeling silk nanoparticles with fluorescent probes

A total of 3.5 mg of 110 and 215 nm silk nanoparticles were fluorescently labeled as follows. First, the respective silk nanoparticles were resuspended in 0.2 M NaHCO_3_ at pH 8.3. Next, either 1 mg of Alexa Fluor 488 succinimidyl ester or Alexa Fluor 594 succinimidyl ester (Life Technologies, Carlsbad, CA, USA) was dissolved in anhydrous DMSO at 1 mg mL^−1^. Then, 100 μL of Alexa Fluor 488 and 100 μL of Alexa Fluor 594 solution were added, respectively, to 110 nm silk nanoparticles and 215 nm silk nanoparticles in 0.2 M NaHCO_3_, pH 8.3. The samples were allowed to react overnight at room temperature in the dark with stirring. The labeled silk nanoparticles were then centrifuged, and the pellets were washed three times with acidified water (pH 4.6) to remove unbound dye, followed by three washes with ultrapure water. The samples were stored at 4 °C in the dark until use.

### Cellular uptake and intracellular distribution of silk nanoparticles

RAW 264.7 cells were seeded and cultured in complete DMEM medium without phenol red. The cells were washed three times with PBS and the culture medium was replaced with either (i) control DMEM or (ii) 0.5 mg mL^−1^ mixed Alexa Fluor 488 (Life Technologies, Carlsbad, CA, U.S.A.) labeled 110 nm silk nanoparticles and Alexa Fluor 594 (Life Technologies, Carlsbad, CA, U.S.A.) labeled 215 nm silk nanoparticles. The cells were either (i) incubated for 1 h or (ii) incubated 1 h followed by three washes with PBS and a 3 h chase in culture medium. The incubation was stopped by placing the cells on ice, aspirating all the medium, and washing three times with ice-cold PBS. The cells were then stained with 1 μg mL^−1^ Hoechst 33342 (Thermo Scientific, Waltham, MA, USA) for 10 min at room temperature in the dark, washed three times with ice-cold PBS, and live cells were imaged immediately with a Leica TCS-SP5 confocal laser scanning microscope (Leica Microsystems GmbH, Wetzlar, Germany) equipped with a 40× magnification water objective with a numerical aperture of 1.25. The data were exported to ImageJ 1.51j8 (National Institute of Health, U.S.A.)^34^ for image analysis and colocalization.

### Statistical analyses

Data were analyzed using GraphPad Prism 7.0 (GraphPad Software, La Jolla, CA, U.S.A.). Sample pairs were analyzed with the Student's *t*-test. Multiple samples were evaluated by One-way and Two-way analysis of variance (ANOVA) followed by Bonferroni's multiple comparison post hoc test or Dunnett's post hoc tests to compare between the control and samples. Asterisks denote statistical significance as follows: *P < 0.05, **P < 0.01, ***P < 0.001. All data are presented as mean values ± standard deviation (SD), and the number of independent experiments (*n*) is noted in each figure legend.

## Results

### The yield of silk nanoparticles

**Fig. 2:**
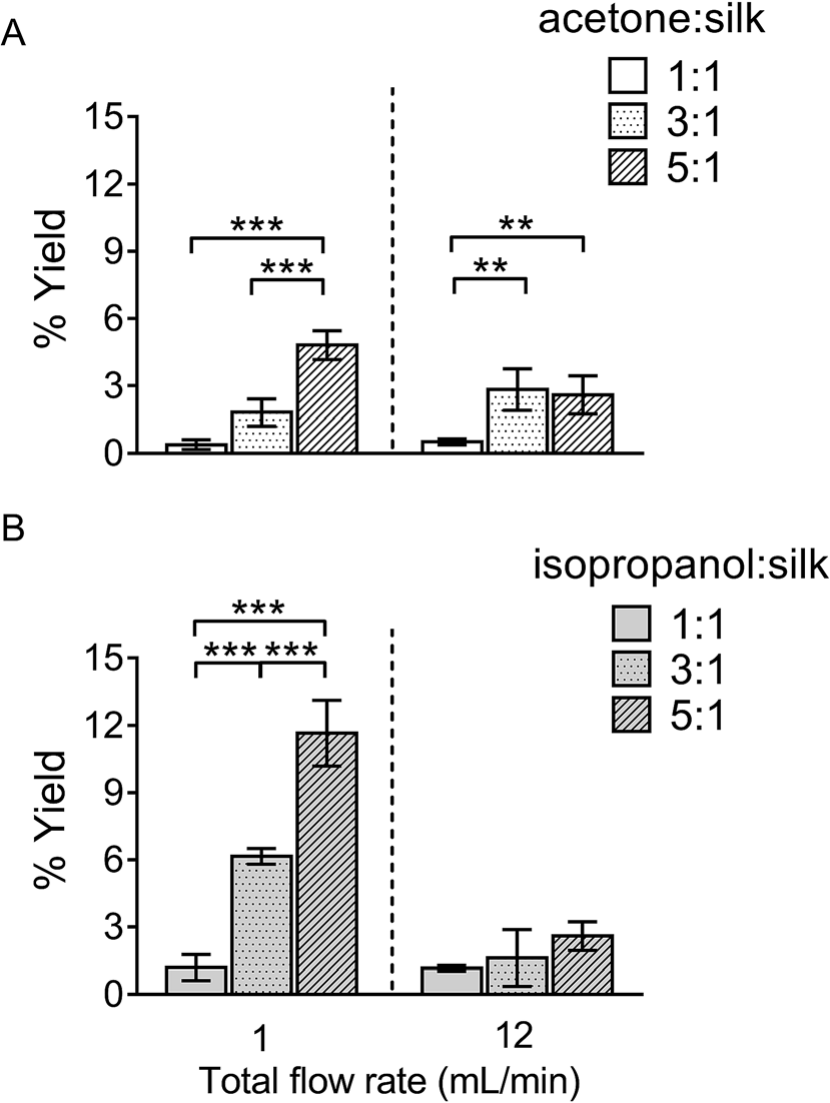
Percentage yield of silk nanoparticles produced with a NanoAssemblr™ benchtop platform by varying the total flow rate and the flow rate ratios. The percentage yield of silk nanoparticles using the organic solvents (A) acetone, and (B) isopropanol. Error bars are hidden in the bar when not visible, ±SD, *n* = 3.

The percentage yield of silk nanoparticles was dependent on the total flow rate, the solvent ratio, and the actual solvent used. A solvent : aqueous total (flow rate) ratio of 5 : 1 gave the best yield for both solvent systems (Fig. 2). The yield of silk nanoparticles was higher when prepared from isopropanol than from acetone, especially at the 1 mL min^−1^ flow rate. The highest silk nanoparticle yield (11.7% w/w of silk) was obtained from isopropanol with the isopropanol : silk (*i.e.*, aqueous phase) ratio of 5 : 1 and a 1 mL min^−1^ flow rate (Fig. 2B).

### Silk nanoparticle characterization and their stability in water

**Fig. 3:**
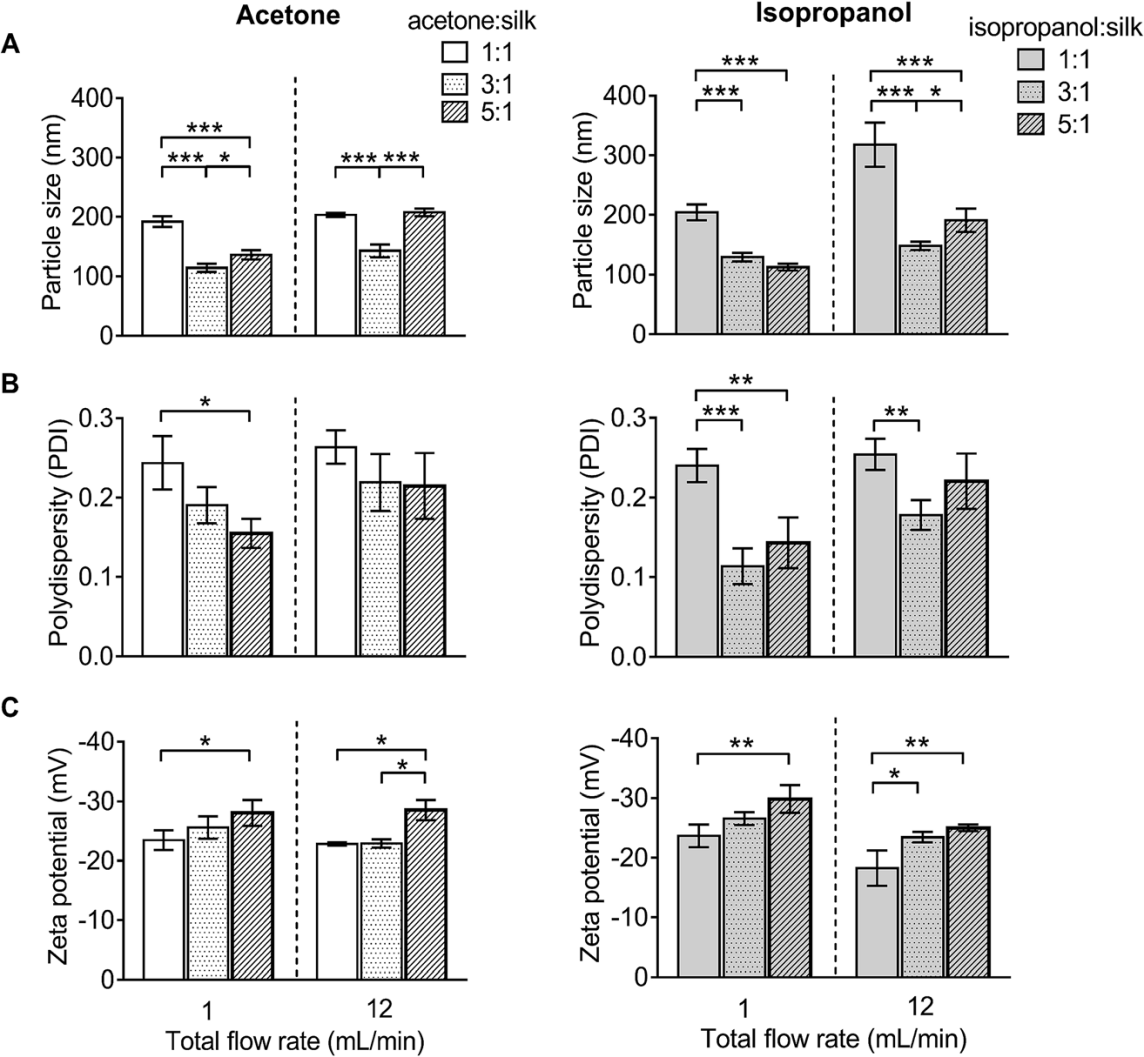
Characterization of silk nanoparticles produced with acetone and isopropanol using a NanoAssemblr™ benchtop platform with different total flow rates and ratios. (A) Particles size (nm), (B) polydispersity index (PDI), and (C) zeta potential of silk nanoparticles. Error bars are hidden in the bars when not visible, ±SD, *n* = 3.

For DLS measurement, the overall particle size of silk nanoparticles ranged from 110 nm to 310 nm, with a polydispersity ranging from 0.1 to 0.25 and a negative surface charge ranging from −20 mV to −30 mV (Fig. 3). The acetone : aqueous total flow rate ratio of 3 : 1 generated the smallest size (110 nm), while the acetone : aqueous total flow rate ratio of 1 : 1 generated larger particles (200 nm) (Fig. 3A). By contrast, the isopropanol : aqueous ratio of 5 : 1 at a 1 mL min^−1^ flow rate generated the smallest size (110 nm) and the isopropanol : aqueous total flow rate ratio of 1 : 1 at 12 mL min^−1^ generated the largest particle size (310 nm) (Fig. 3A). However, a solvent : aqueous ratio of 1 : 1 showed higher polydispersity (>0.2), indicative of a wider particle size distribution (Fig. 3B). The solvent : aqueous total flow rate ratio of 5 : 1 generated higher negative charges of silk nanoparticles when compared with a lower ratio of solvents (Fig. 3C).

**Fig. 4:**
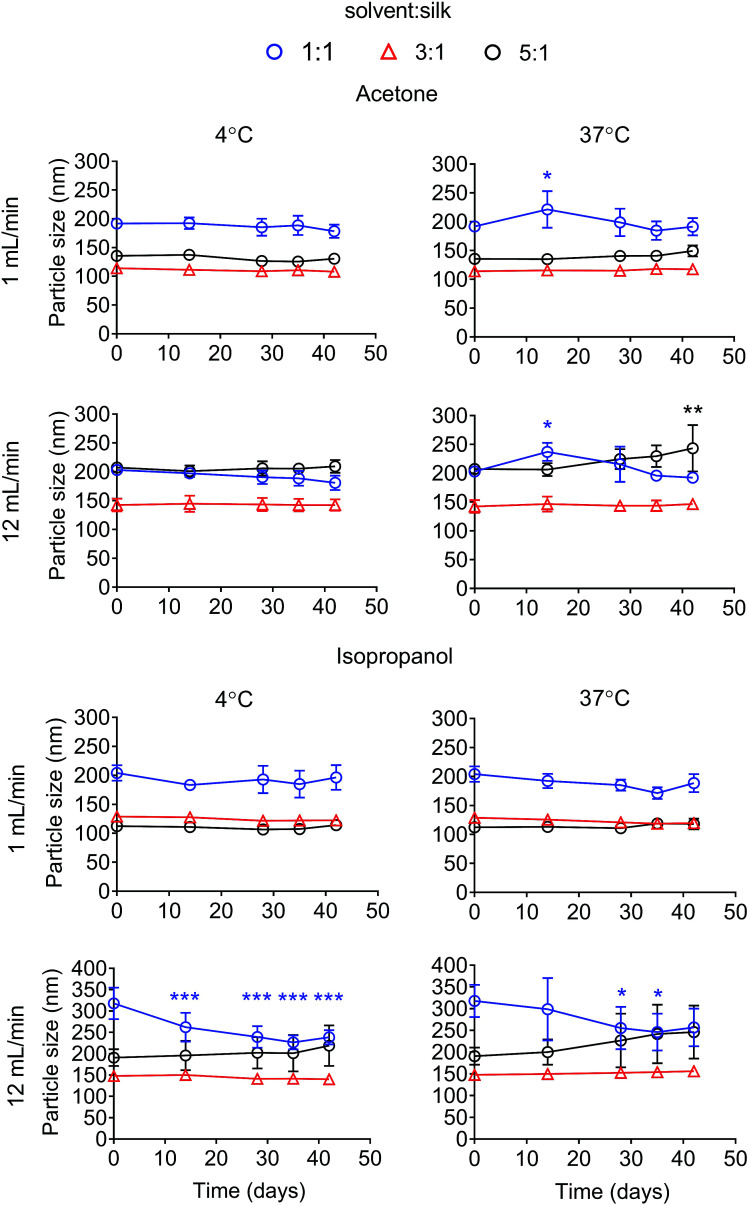
Stability of silk nanoparticles manufactured with a microfluidic-based method by varying solvents, the total flow rate, and the flow rate ratios. The particle size of the silk nanoparticles in water at 4 °C and 37 °C was measured over 42 days. Error bars are hidden in the plot symbols when not visible, ±SD, *n* = 3.

The particle size stability was also determined for up to 42 days. For the acetone system, all formulations were stable in water at 4 °C for up to 42 days. Silk nanoparticles generated with a ratio of solvent to silk of 5 : 1 at 12 mL min^−1^ showed a statistical significant increase in particle size after storage at 37 °C for 42 days (Fig. 4). The polydispersity of the silk nanoparticles did not change at 4 °C and 37 °C for up to 42 days (Fig. S1†). For the isopropanol system, silk nanoparticles generated with the ratios of solvent : silk of 3 : 1 and 5 : 1 were stable at 4 °C and 37 °C for up to 42 days. However, silk nanoparticles generated with a isopropanol : silk flow rate ratio of 1 : 1, especially at the total flow rate of 12 mL min^−1^, were not stable after 14 days (Fig. 4). The polydispersity of the silk nanoparticles from an isopropanol : silk flow rate ratio of 5 : 1 slightly increased after 28 days (Fig. S1†). The negative surface charges of the silk nanoparticles from all formulations significantly decreased after 14 days at 37 °C (Fig. S2†).

### Secondary structure measurement

**Fig. 5:**
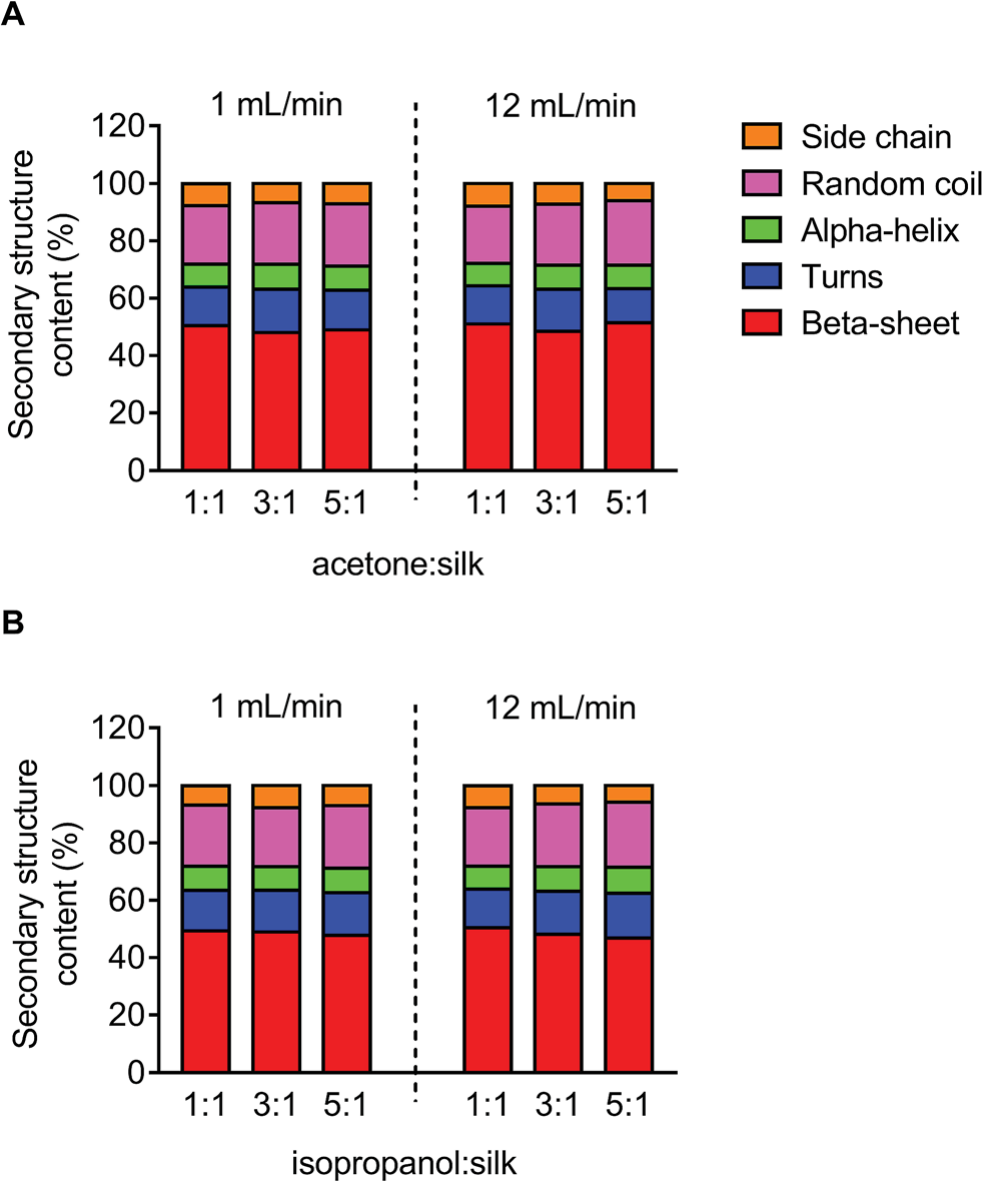
Secondary structure analysis of silk nanoparticles produced by microfluidics by varying the total flow rate, flow rate ratio, and solvents. Secondary structure content (%) of silk nanoparticles using (A) acetone and (B) isopropanol as the organic solvent.

The secondary structure of the silk nanoparticles produced under different process conditions was determined by FTIR measurement following peak analysis. Overall, silk nanoparticles manufactured using microfluidics had a high beta-sheet content (48–51%), and changes in the microfluidic parameters had no significant effect on this content (Fig. 5).

### Scanning electron microscope of silk nanoparticles

**Fig. 6:**
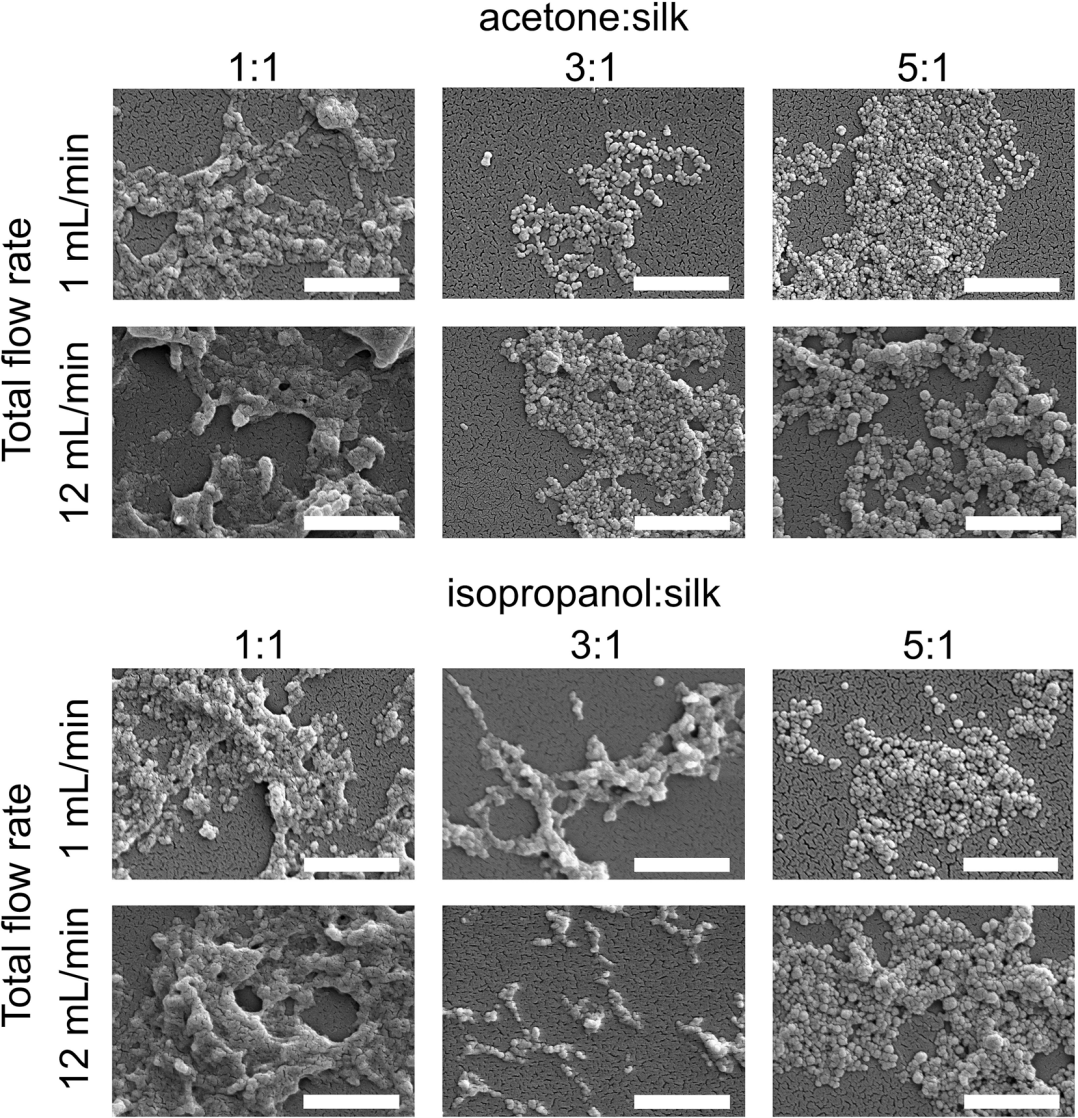
Scanning electron microscopy images of silk nanoparticles produced using the NanoAssemblr™ benchtop platform with different total flow rates and ratios of organic solvents (scale bar = 1 μm).

The morphology of silk nanoparticles was analyzed by SEM (Fig. 6). The silk nanoparticles generated by the solvent : aqueous total flow rate ratios of 3 : 1 and 5 : 1 had spherical shapes and uniform distributions, which correlated with the DLS measurements. Silk nanoparticles obtained using a total flow rate ratio of 1 : 1, especially at the total flow rate of 12 mL min^−1^, showed larger sizes (up to 400 nm), irregular shapes, and wide particle distributions (particles ranging from 200 nm to 400 nm) (Fig. 6).

### *In vitro* cytotoxicity and macrophage responses to silk nanoparticles

**Fig. 7:**
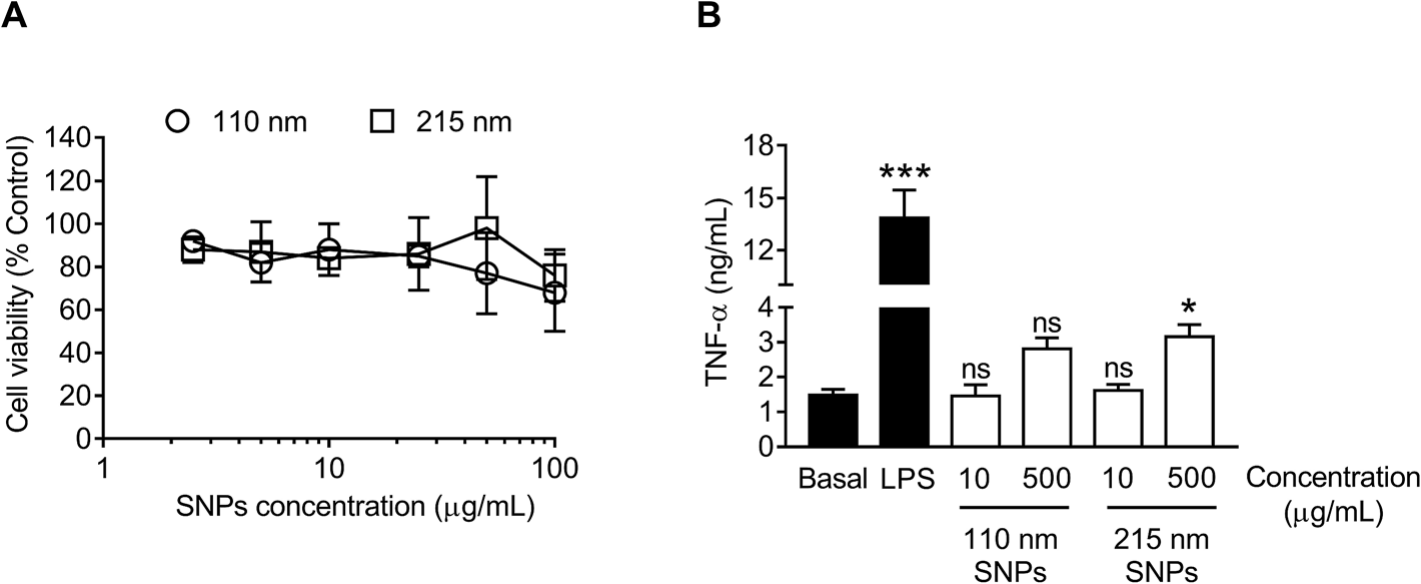
*In vitro* cytotoxicity and TNF-α release from macrophages (RAW 264.7 cells) in response to treatment with silk nanoparticles. (A) For cytotoxicity tests, 2.5 to 100 μg mL^−1^ of 110 nm and 215 nm silk nanoparticles (SNPs) were incubated with the cells for 48 h. (B) The TNF-α release into culture supernatants was quantified following a 24 h of exposure to 15 ng of LPS (positive control) or 10 μg mL^−1^ and 500 μg mL^−1^ of 110 nm and 215 nm silk nanoparticles and compared to release by untreated control cells (basal TNF-α levels). Dunnett's post hoc test was used to evaluate statistical differences between the basal and the samples. Error bars are hidden in the plot-symbol when not visible, ±SD, *n* = 3.

For cytotoxicity studies, two different sizes of silk nanoparticles (110 and 215 nm) were generated. No significant differences were noted in cytotoxicity between the two different sizes of silk nanoparticles (Fig. 7A). The half maximal inhibitory concentration (IC50) of 110 nm and 215 nm silk nanoparticles toward RAW 264.7 cells was >100 μg mL^−1^. The TNF-α release by macrophages exposed to silk nanoparticles and LPS (positive control) was also measured (Fig. 7B). TNF-α release in response to 110 nm silk nanoparticles (at both 10 μg mL^−1^ and 500 μg mL^−1^) did not differ significantly from the release by control cultures. However, treatment of the cells with 500 μg mL^−1^ of 215 nm silk nanoparticles caused a small, but statistically significant, increase in TNF-α release when compared to 110 nm nanoparticles at the equivalent dose (Fig. 7B). There was a statistical difference TNF-α release between concentration of 10 μg mL^−1^ and 500 μg mL^−1^ of the silk nanoparticles (Student's *t*-test).

### Cellular uptake and intracellular distribution of silk nanoparticles in macrophages

**Fig. 8:**
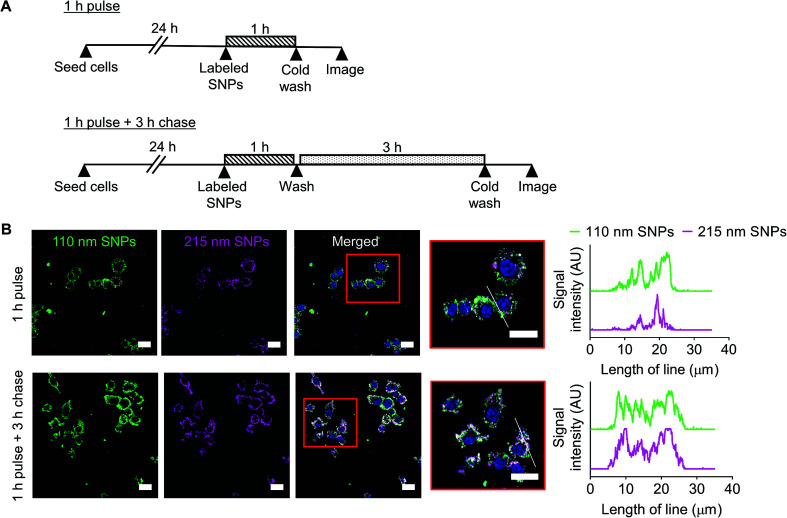
Impact of silk nanoparticle size on trafficking in macrophages. (A) Experimental outline. Cells were either (i) pulsed with a mixture of 110 nm and 215 nm labeled silk nanoparticles for 1 h and imaged or (ii) pulsed with the labeled silk nanoparticles for 1 h and then chased for 3 h and imaged. (B) Live cell confocal fluorescence microscopy of the mixture of Alexa Fluor 488-labeled 110 nm silk nanoparticles (green) and Alexa Fluor 594-labeled 215 nm silk nanoparticles (magenta) in RAW 264.7 cells. The scale bars are 20 μm. The white lines in the high magnification images are used in profile plots to highlight the colocalization of 110 nm and 215 nm silk nanoparticles in the cells.

Cellular uptake and intracellular distribution of 110 nm and 215 nm silk nanoparticles were qualitatively studied using live-cell confocal microscopy (Fig. 8). Following a 1 h pulse, 110 nm silk nanoparticles and 215 nm silk nanoparticles were internalized into different early endosome compartments. However, after a 3 h chase, both sizes of silk nanoparticles were localized in the same late endocytic compartments. These results were corroborated by profile plots that showed high co-localization after the 3 h chase (Fig. 8).

## Discussion

Silk from *Bombyx mori* has a strong clinical track record^1^ and is currently emerging as a promising biomaterial for drug delivery (*e.g.*, ref. 5, 6 and 35). The manufacture of silk nanoparticles is now increasingly reported (typically in the 100 nm size range), often for (anticancer) drug delivery applications (*e.g.*, ref. 10 and 36). Our use of silk nanoparticles has specifically focused on drug loading and release,^7^ surface modification,^8^ intracellular drug delivery,^9^ and the degradation of silk nanoparticles in cells,^12^ as well as on the impact of these nanoparticles on metabolism and blood compatability.^11,37^ However, in all of these previous studies, we used 100 nm silk nanoparticles that we fabricated using a “conventional” nanoprecipitation method; *i.e.*, manually adding the reverse-engineered silk solution to the organic phase.^25^ This production method is a batch-based process and affords no in-process control to fine tune the particle properties. Therefore, a manufacturing method that enables rapid silk nanoparticle production while providing control over the nanoparticle characteristics would represent a substantial improvement and would open up the use of these silk nanoparticles in a wider spectrum of biomedical applications.

Microfluidic-based technologies have been successfully used for liposome and nanoparticle production (*e.g.*, using 1,2-dipalmitoylphosphatidylcholine, phosphatidylcholine, polycaprolactone-*block*-poly(ethylene oxide) and poly(lactide-*co*-glycolide)-*b*-polyethyleneglycol), which allow scalable production and control over the particle characteristics.^38–41^ Many different microfluidic platform designs have been introduced, but the most important feature is the mixer channel layout, which has included, for example, droplet based^42^ as well as T and Y shaped mixers.^43,44^ The staggered herringbone structure is a highly efficient micromixer and is now one of the commonest designs, as it enhances the mixing of the aqueous and solvent phases due to chaotic advection phenomena.^45^ The staggered herringbone micromixer is the most efficient passive mixer and could therefore be regarded as a “three dimensional” mixer. The staggered herringbone design shows higher mixing efficiency when the Reynolds numbers is in a range of 0 < *N*_Re_ < 1000 (low *N*_Re_). Therefore, mixing efficiency declined as Reynolds number increased with increasing flow rate. In the present study, we used the fully automated NanoAssemblr™ platform in combination with a commercially available microfluidic chip that incorporates the staggered herringbone structure design (Fig. 1).

We believe that this study is the first to report the continuous manufacture of silk nanoparticles. The production efficiency for generation of silk nanoparticles showed that the optimal conditions for achieving the highest silk nanoparticle yields were a total flow rate at 1 mL min^−1^ at a 5 : 1 solvent : aqueous ratio (Fig. 2). We speculate that this slower total flow rate (*versus* 12 mL min^−1^) and high solvent concentration allows more time for interaction between the aqueous and solvent phases, thereby enabling a better removal of solvating water from the silk structure and ultimately resulting in silk nanoparticle formation through beta-sheet formation.^46^ Therefore, solvents with a high capacity to form hydrogen bonds with water are predicted to be good candidates for silk nanoparticle formation. We therefore selected isopropanol (a polar protic organic solvent), as it has a greater ability than acetone (or DMSO) (a polar aprotic organic solvents) to form hydrogen bonds with water. Previous batch-based studies have also successfully used isopropanol for silk nanoparticle formation (particle size ranging from 100 to 400 nm).^46,47^ In the current study, the choice of a low flow rate of 1 mL min^−1^, the selection of isopropanol, and the use of a high organic solvent : aqueous ratio (*i.e.*, 5 : 1) led to significant improvements in yield (Fig. 2). Both the solvent : aqueous ratio and the flow rate had a significant impact on the particle size, PDI, and zeta potential (Fig. 3). The high solvent : aqueous ratio (*i.e.*, ≥ 3 : 1) generated small particles (110–200 nm) with a low polydispersity index (0.1–0.2) and high negative surface charge (−23 to −30 mV). Overall, these data highlight the importance of using sufficient amounts of solvent to extract the solvating water from the silk to initiate uniform nanoparticle nucleation and, ultimately, to narrow the particle size distribution. The zeta potential of silk nanoparticles produced using the microfluidic setup was less negative (−20 to −30 mV) when compared to those produced by a standard batch method (−40 mV to −50 mV, *e.g.*, ref. 8 and 9). This comparatively low negative zeta potential could be a consequence of the continuous flow during particle formation, which could ultimately result in a different packing arrangement.

We also examined silk nanoparticle stability in water over 42 days, because (medical) applications of these silk nanoparticles requires them to have long-term stability during storage (Fig. 4, S1 and S2†). Silk nanoparticles generated from microfluidics using a solvent : aqueous total flow rate ratio ≥ 3 : 1 were stable at 4 °C and 37 °C over the entire study period. This finding confirms the importance of the desolvating solvent concentration for silk nanoparticle formation and stability, because low solvent to silk concentration ratios resulted in nanoparticles with compromised stability. We therefore also expect to see differences in secondary structure, because silk nanoparticles with a low beta-sheet content have been reported.^48^ However, the silk nanoparticles prepared by microfluidics had a comparable beta-sheet content (Fig. 5), indicating that this content was independent of the process parameters. Overall, all the silk nanoparticles generated were highly crystalline and essentially identical with respect to their secondary structure to nanoparticles we have previously reported.^8,9^

Morphological assessment by electron microscopy indicated that the total flow rate and the flow rate ratio were the key parameters that influenced the particle appearance (Fig. 6). Silk nanoparticles generated with a slow flow rate (1 mL min^−1^) showed a more globular shape and appeared as discrete nanoparticles when compared with those generated using a flow rate of 12 mL min^−1^, suggesting that the fast flow rate could disrupt the spherical morphology during particle formation and result in a greater tendency of these particles to undergo a loose fusion. Due to the high molecular weight of the biopolymer silk (390 kDa), silk nanoparticle formation requires a sufficient amount of organic solvent for water removal in order to form packed silk nanoparticles. However, at a high total flow rate (*i.e.* 12 mL min^−1^), one might speculate there was not enough time for efficient mixing of the two phases resulting in lower water removal. This in turn could results in “loosely” packed silk nanoparticles as evidenced by their irregular shape (Fig. 6) and low yield (Fig. 2). Overall, achieving a more discrete globular shape and uniformity required a solvent : aqueous flow rate ratio ≥ 3 : 1 (and a slow flow rate). This minimum solvent to water ratio for the formation of silk nanoparticles is consistent with previous batch-based silk particle work.^47^

The nanosize range of silk nanoparticles is expected to result in solid tumor targeting in medical applications because the passive accumulation of nanoparticles (*e.g.*, 100 to 200 nm) is facilitated by the tumor pathophysiology, which includes a leaky vasculature and impaired lymphatic clearance that results in enhanced permeation and retention (EPR) of nanomedicines.^49^ However, even EPR-mediated targeting typically results in only a small fraction of the administered dose reaching the tumor,^50^ with most medicine accumulating in other tissues, predominantly in macrophages of the mononuclear phagocytic system.^51^ Macrophages are intimately associated with solid tumor development;^52^ therefore, the macrophage response toward nanomedicines is an important consideration. We have previously demonstrated that silk nanoparticles can prime macrophages toward an M1-like phenotype.^37^ Emerging evidence indicates that nanoparticle size is important for macrophage recognition and subsequent particle internalization.^53^ We therefore examined the relationship of silk nanoparticle size to cytotoxicity, TNF-α release, cellular uptake, and intracellular distribution. Cytotoxicity was absent at the doses studied (*i.e.*, keeping the amount of silk constant), with no obvious size-dependent cytotoxicity (Fig. 7A). We then selected low and high doses of 110 nm and 215 nm silk nanoparticles and monitored TNF-α release. At the maximum tested concentration, only a small increase was noted, but a statistically significant difference in TNF-α release was observed for 215 nm silk nanoparticles when compared to 110 nm particles (Fig. 7B). Nevertheless, the biological relevance of this difference is currently not known. Preliminary intracellular trafficking studies showed that 110 nm and 215 nm silk nanoparticles were both internalized by endocytosis within 1 h, but they were localized into different early endocytic structures. Following a 3 h chase, the silk nanoparticles of both sizes accumulated in late endosomal/lysosomal compartments, as suggested by their peri-nuclear localization (Fig. 8). The observed differences in trafficking at the early time point could suggest that endocytic compartments were size-selective, as reported previously for labeled erythrocytes.^54^ However, more detailed studies are needed to better characterize the intracellular trafficking of silk nanoparticles.

## Conclusions

The use of a microfluidic setup enabled the rapid, reproducible and controllable manufacture of silk nanoparticles. The total flow rate and the flow rate ratio were the two key process parameters that affected silk nanoparticle characteristics. A total flow rate of 1 mL min^−1^ and a solvent to aqueous phase ratio of 5 : 1 provided the smallest particle size, the highest yield, and best stability of silk nanoparticles. Subjecting the optimized silk nanoparticles to preliminary biological assessment indicated that they induced a particle-mediated macrophage response. In summary, microfluidic-assisted manufacturing enables the fine tuning of silk nanoparticles.

## Author contributions

T. W. acquired, analyzed, and interpreted data, and generated the manuscript draft. J. D. T. designed and performed confocal microscopy studies. All authors (T. W., J. D. T., B. F. J., and F. P. S.) designed research, discussed the results, and/or advised on the analysis. F. P. S. conceived the study and edited the manuscript with support from the other authors.

## Conflicts of interest

There are no conflicts to declare.

## Supplementary Material

NA-001-C8NA00208H-s001
