# Supplementary material for: Microfluidic-assisted silk nanoparticle tuning
Source: Nanoscale Adv. 2018 Nov 30;1(2):873–83. doi: 10.1039/c8na00208h (PMC9473249; doi:10.1039/c8na00208h)
Supplement: NA-001-C8NA00208H-s001 [file NA-001-C8NA00208H-s001.pdf]

Electronic Supplementary Material (ESI) for Nanoscale Advances.
This journal is © The Royal Society of Chemistry 2018

Supporting Information

Microfluidic-assisted silk nanoparticle tuning

Thidarat Wongpinyochit [1], John D. Totten[1], Blair F. Johnston [1], F. Philipp Seib [1,2,]*

[1] Strathclyde Institute of Pharmacy and Biomedical Sciences, University of Strathclyde, 161 Cathedral Street, Glasgow, G4 0RE, UK. E-mail: philipp.seib@strath.ac.uk

[2] Leibniz Institute of Polymer Research Dresden, Max Bergmann Center of Biomaterials Dresden, Hohe Strasse 6, 01069 Dresden, Germany

* Corresponding author: Tel. +44 (0) 141 548 2510, E-mail: philipp.seib@strath.ac.uk or philipp.seib@SeibLab.com

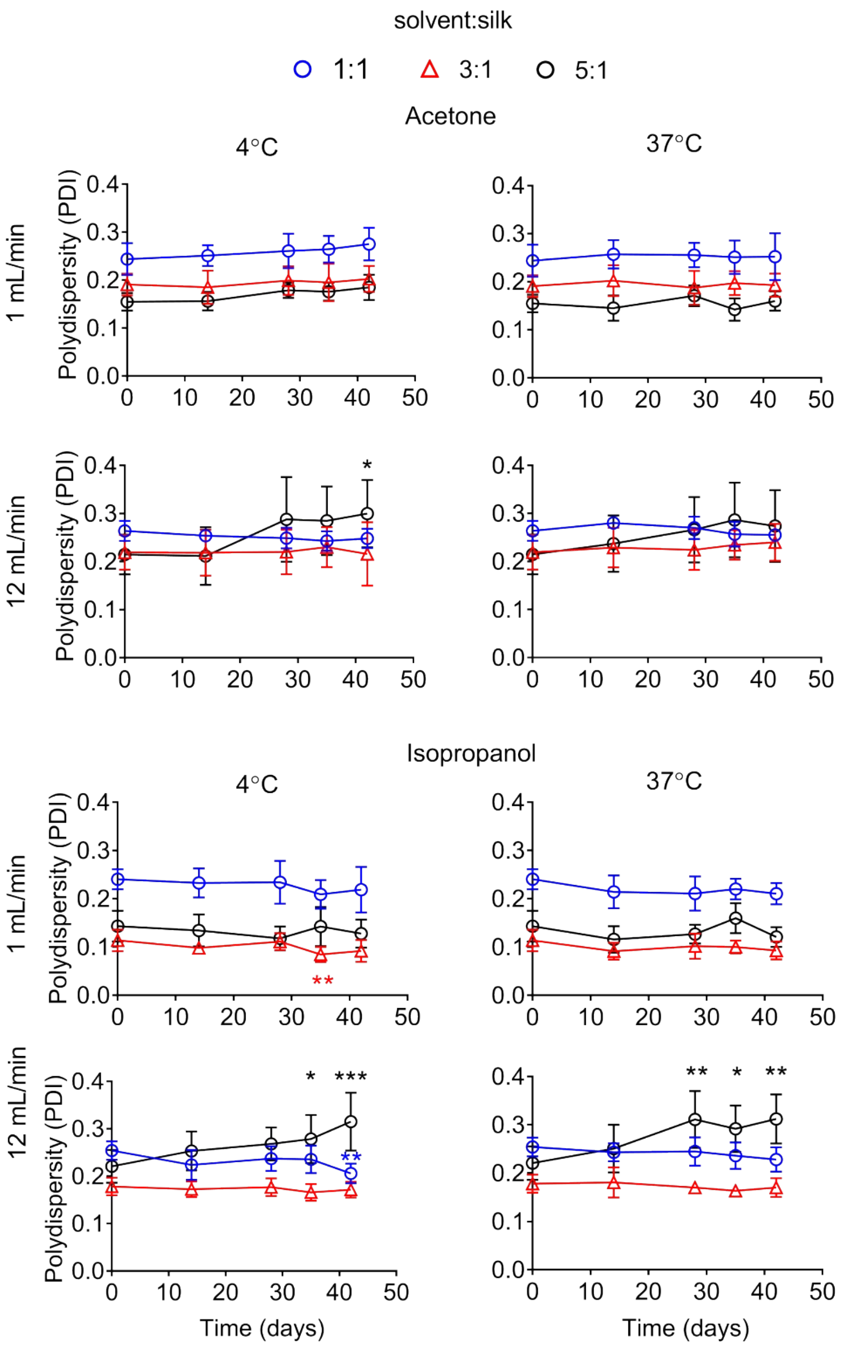

Figure S1 Stability of silk nanoparticles manufactured with a microfluidic-based method by varying solvents, the total flow rate and the total flow rate ratio. Polydispersity (PDI) of silk nanoparticles in water at 4°C and 37°C was measured at day 0, 14, 28, 35 and 42. One-way ANOVA followed by Bonferroni's multiple comparison post hoc test, *P < 0.05, **P < 0.01, *** P < 0.001. Error bars are hidden in the plot -symbol when not visible, ±SD, n = 3.

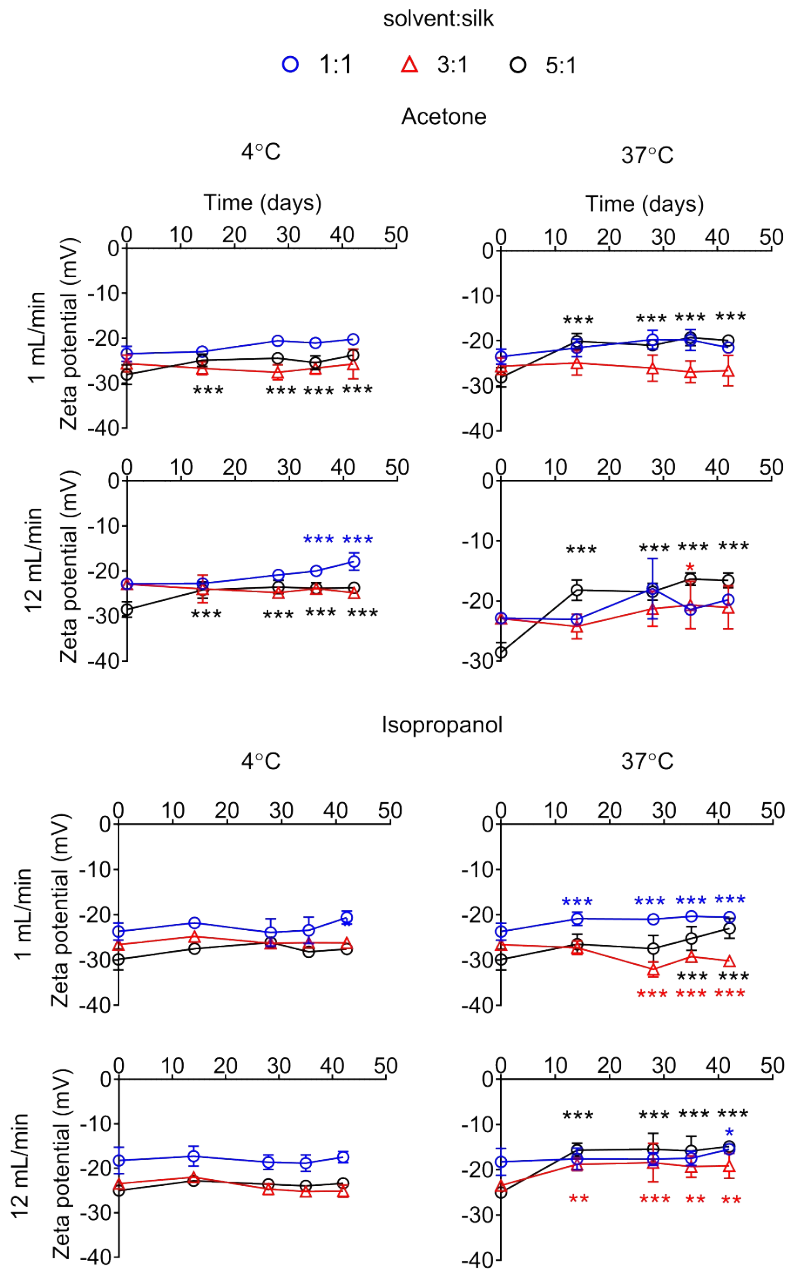

Figure S2 Stability of silk nanoparticles manufactured with a microfluidic-based method by varying solvents, the total flow rate and the total flow rate ratio. Zeta potential of silk nanoparticles in water at 4°C and 37°C was measured at day 0, 14, 28, 35 and 42. One-way ANOVA followed by Bonferroni's multiple comparison post hoc test, *P < 0.05, **P < 0.01, *** P < 0.001. Error bars are hidden in the plot -symbol when not visible, ±SD, n = 3.
